# A multicenter, phase 1, dose escalation clinical trial (G-FORCE-1) of XRT, RRx-001 and temozolomide followed by temozolomide +/- RRx-001 in newly diagnosed glioblastoma

**DOI:** 10.3389/fonc.2023.1176448

**Published:** 2023-06-05

**Authors:** Howard Fine, Tony Reid, Scott Caroen, Bryan Oronsky, Nacer Abrouk, Nicholas Butowski

**Affiliations:** ^1^ Department of Neuro-oncology, Brain Tumor Center at New York-Presbyterian Weill Cornell Medical Center, New York, NY, United States; ^2^ Department of Oncology, EpicentRx, Torrey Pines, CA, United States; ^3^ Department of Drug Development, Clinical Trial Innovations, Mountain View, CA, United States; ^4^ Department of Neuro-oncology, UCSF Brain Tumor Center, San Francisco, CA, United States

**Keywords:** RRx-001, primary GBM, brain penetrant, hypoxia activated, radiotherapy, temozolomide (not in MeSH)

## Abstract

**Introduction:**

The current standard of care for newly diagnosed glioblastoma (GBM) is maximum surgical resection followed by concurrent treatment with temozolomide (TMZ) and radiotherapy (RT) and then six to twelve cycles of maintenance TMZ. RRx-001, an NLRP3 inhibitor and nitric oxide (NO) donor with chemoradiosensitizing, vascular normalizing and macrophage repolarizing properties, is currently in a Phase III trial for small cell lung cancer (SCLC). The purpose of this non-randomized trial was to establish the safety and look for a signal of clinical activity of RRx-001 as an add-on to RT and TMZ in patients with newly diagnosed glioblastoma.

**Methods:**

In this non-randomized, open-label, two part trial called G-FORCE-1 (NCT02871843), the first four cohorts of adult patients with histologically confirmed high grade gliomas received fractionated radiotherapy (60 Gy in 30 fractions over 6 weeks), daily 75 mg/m2 temozolomide and escalating doses of once weekly RRx-001 from 0.5 mg to 4 mg according to a 3+3 design followed by a 6 week no treatment interval and then standard maintenance TMZ (150 mg/m2 Cycle 1 and 200 mg/m2 in subsequent cycles) until disease progression. The second two cohorts of patients received fractionated radiotherapy (60 Gy in 30 fractions over 6 weeks), daily 75 mg/m2 temozolomide and once weekly RRx-001 4 mg followed by a 6 week no treatment interval and then two different maintenance schedules until disease progression according to the same 3+ 3 design: 1. 0.5 mg RRx-001 once weekly + 100 mg/m2 TMZ 5 days/week for up to 6 cycles of therapy; 2. 4 mg RRx-001 once weekly + 100 mg/m2 TMZ 5 days/week for up to 6 cycles of therapy

The primary endpoint was the recommended dose/maximally tolerated dose of the combination of RRx-001, TMZ and RT. Secondary endpoints were overall survival, progression free survival, objective response rate, duration of response and clinical benefit response.

**Results:**

A total of 16 newly diagnosed glioblastoma patients were enrolled. No dose limiting toxicities were observed and no MTD was reached. The recommended dose is 4 mg. After 24 months of follow up the median OS was 21.9 months (95% CI: 11.7 – NA). PFS median was 8 months (95% CI: 5 – NA). The overall response rate was 18.8% (3 PR out of 16) and the disease control rate was 68.8% (3 PR, 8 SD out of 16).

**Conclusions:**

The addition of RRx-001 to TMZ and RT and to TMZ during maintenance was safe and well-tolerated and deserves further study.

## Highlights

- RRx-001 is a novel, first-in-class molecule and, to best knowledge, the only NLRP3 inhibitor that has been evaluated clinically in GBM
- It was evaluated in a Phase 1 trial for first line GBM patients as an add on to radiation and temozolomide
- RRx-001 plus temozolomide and radiotherapy was safe and well tolerated
- Preliminary evidence of efficacy potential was observed

## Importance of the study

RRx-001 is a brain penetrant small molecule with Food and Drug Administration (FDA) Fast Track designation in head and neck cancer that donates nitric oxide under hypoxia, polarizes tumor associated macrophages, and normalizes the tumor vasculature. G-FORCE-1 is the first clinical trial to evaluate RRx-001 as an add-on therapy to temozolomide and radiotherapy in first line GBM. This study determined the recommended Phase 2 dose of RRx-001 given once weekly as flat 4 mg dose and once weekly during temozolomide maintenance. The combination was safe and well tolerated and preliminary evidence of activity was observed. To best knowledge, RRx-001 is the only NLRP3 inhibitor that has been evaluated clinically in GBM.

## Introduction

Glioblastoma, despite a relatively rare incidence of 12,000 per year (1) in the United States, is the most common primary malignant brain tumor in adults. The standard of care, RT with concomitant temozolomide (TMZ) followed by six to twelve cycles of adjuvant TMZ, is based on a phase III trial in which the median overall survival (OS) of patients that underwent this regimen after surgical resection was 14.6 months vs. 12.1 months for RT alone (2). The prognosis of GBM is uniformly dismal, despite the addition of TMZ to RT, with few patients surviving beyond 5 years, and, therefore, more effective regimens are urgently needed. These poor outcomes are related to several factors including perturbed drug delivery secondary to the blood brain barrier and abnormal tumor-associated angiogenesis, intrinsic tumor radiation and chemotherapy resistance as a function of tumor genetic and epigenetic clonal heterogeneity, the diffuse infiltrative nature of the tumor and the sensitivity microenvironment of the brain. A strategy, which can overcome one or more of these factors, has the potential to improve the antitumor effect of RT and TMZ for GBM patients.

RRx-001 (3) is a minimally toxic Nod-like receptor family pyrin domain containing 3 (NLRP3) inhibitor and nitric oxide (NO) donor with chemoradiosensitizing, vascular normalizing, and macrophage repolarizing properties (4, 5) in a Phase 3 trial called REPLATINUM (NCT03699956) for the treatment of small cell lung cancer (SCLC). It has been shown to cross the blood brain barrier (6) and appears both clinically (7, 8) and preclinically to reverse multidrug resistance as well as to improve the delivery of chemotherapy and oxygen *via* vascular normalization (9) and to treat several neurodegenerative diseases such as Alzheimer’s (10), Parkinson’s, amyotrophic lateral sclerosis (ALS)/motor neuron disease (MND), and multiple sclerosis (11). In over 300 patients, no serious adverse events have been attributed to RRx-001. In preclinical studies, RRx-001 demonstrated single agent activity in temozolomide-sensitive and resistant GBM (12). Additionally, increased intratumoral uptake of TMZ as well as improved anticancer activity compared to TMZ as a single agent was demonstrated in glioma-bearing mice that were treated with RRx-001 + TMZ (13). On the basis of these preclinical experiments as well as the chemoradiosensitizing potential of RRx-001 in other clinical studies, the G-FORCE-1 study (NCT02871843) was conducted to test the safety of RRx-001, firstly, when combined with standard TMZ and RT dosing in high-grade glioma patients and, secondly, when combined with TMZ maintenance. In addition, the excessive activation of the NLRP3 inflammasome leads to chronic inflammation through overexpression of the proinflammatory cytokines interleukin (IL)-1β and interleukin (IL)-18; in turn, chronic inflammation is associated with tumorigenesis and progression as well as immune evasion in several cancer types including human gliomas (14, 15). Therefore, as an NLRP3 inhibitor, which has been funded by the Michael J. Fox Foundation and FightMND to study RRx-001 in Parkinson’s disease and amyotrophic lateral sclerosis (ALS)/motor neuron disease (MND), IL-1β and IL-18 are potential biomarkers of activity of RRx-001 in GBM; however, as this was, first and foremost, a safety and tolerability trial, IL-1β and IL-18 levels were not measured. It is unclear whether, unlike RRx-001, NLRP3 agonists, which increase plasma levels of pro-inflammatory cytokines such as IL-1β, may, under certain circumstances, for example, in the case of nonimmunogenic or “cold” tumors, also have anticancer activity.

## Methods

### Study patients

Eligible patients were at least 18 years of age with newly diagnosed high grade gliomas and no other concurrent treatment. Additional eligibility criteria included a Karnofsky performance status of at least 70 and adequate hematologic, renal, and hepatic function. In addition, patients were required to undergo treatment between 3-6 weeks after surgery or biopsy. Exclusion criteria included: prior invasive malignancy, recurrent disease, infratentorial spread, prior chemotherapy or chemotherapy for the head and neck, active connective tissue disorders due to the risk of radiation toxicity. The MGMT promoter methylation status and IDH mutation status were retrieved from the clinical records at each site. All patients provided written informed consent. The study was approved by the institutional review board at each center before patient enrollment.

### Study treatment

Fractionated, conformal radiotherapy or intensity-modulated radiotherapy (IMRT) was given at a daily dose of 2 Gy. Treatment was delivered 5 days a week for 6 weeks to an initial volume consisting of the contrast-enhancing tumor plus surrounding edema and a 2-cm margin, for a total dose of 46 Gy in 23 fractions, followed by a conedown or boost of 14 Gy in 7 fractions to the contrast-enhancing tumor and a 2.5-cm margin (cumulative dose of 60 Gy in 30 fractions).

Treatment with temozolomide, at a dose of 75 mg per square meter of body-surface area, was started at the initiation of radiotherapy and was continued daily until the completion of radiotherapy, with a maximum of 49 doses, along with once weekly RRx-001 in escalating doses of 0.5 mg, 1 mg, 2 mg, and 4 mg for a maximum of 6 doses in the first four cohorts of patients.

Maintenance treatment with temozolomide began 6 weeks after the completion of radiotherapy at a starting dose of 150 mg per meter squared for 5 consecutive days of a 28-day cycle, with an increase to 200 mg per square meter for subsequent cycles if no treatment-related adverse events of grade 2 or higher were observed. Treatment was planned for 6 cycles. Antiemetic therapy with the use of a 5-hydroxytryptamine receptor antagonist was strongly recommended. Pneumocystis prophylaxis was at the discretion of the Investigator.

In the 5^th^ and 6^th^ cohorts, patients also received concomitant TMZ at a dose of 75 mg/m^2^ daily during radiation treatment but this time with a flat 4 mg weekly dose of RRx-001. For the maintenance portion, which also began 6 weeks after the completion of radiotherapy, the 5^th^ cohort received 0.5 mg RRx-001 given once weekly with 100 mg/m^2^ of TMZ for 5 consecutive days until progression or until the completion of 6 cycles, whichever came first while the 6^th^ cohort received 4 mg RRx-001 given once weekly with 100 mg/m^2^ of TMZ for 5 consecutive days until progression or until the completion of 6 cycles, whichever came first.

### Patient evaluation and follow-up

At baseline, all patients underwent a physical examination that included a neurologic assessment, complete blood counts, blood chemical analyses (including tests of renal and hepatic function), and tumor imaging with either MRI (preferred) or CT, as well as a serum pregnancy test in women of child-bearing age. During radiotherapy, patients were assessed for adverse events weekly and underwent weekly complete blood counts blood chemical analyses. During the maintenance phase of treatment, patients underwent blood counts and blood chemical analyses on day 1 of each 28-day cycle.

A repeat tumor-imaging study was performed approximately 4 weeks after completion of radiotherapy and then every 8 weeks until tumor progression. Response was assessed *via* modified RANO criteria (16) which defined progression as an increase in tumor size by at least 25% or the development of a new lesion. Since pseudoprogression is common in GBM, whereby tumors transiently enlarge or new lesions appear and then regress, investigators were encouraged not to declare tumor progression within the first 12 weeks after completion of radiotherapy unless clear evidence of progression outside of the radiation field was present or a new lesion or neurologic worsening occurred. Toxicity was recorded and graded according to the National Cancer Institute Common Terminology Criteria for Adverse Events (CTCAE), version 5.0.

### Primary and secondary end points

The primary endpoint was the recommended dose/maximally tolerated dose of the combination of RRx-001, TMZ and RT. Secondary endpoints were overall survival, which was defined as the time until death from any cause, progression free survival, which was defined as the time until either disease progression or death, objective response rate, duration of response and clinical benefit response.

### Study oversight

All treatment data were collected by the study sponsor, EpicentRx, and reviewed by all the authors. The analyses were performed by an independent statistician. All authors reviewed and approved the manuscript. They confirm their adherence to the study protocol and the completeness and accuracy of the data.

### Statistical analysis

Data analysis was based on the intention-to-treat population comprising all enrolled subjects. Overall survival was analyzed using the Kaplan-Meier method and estimates of the survival curve and its median were derived (with the corresponding 95% confidence interval). The employed censoring mechanism followed the standard convention (subjects with unobserved events at the time of the analysis were right censored). The analysis of progression-free survival was similar. Safety data summary of adverse events employed standard MedDRA coding and was presented in terms of sample size and frequency of the number of patients who experienced adverse events or severe adverse events.

### Study treatment

**Figure 1:**
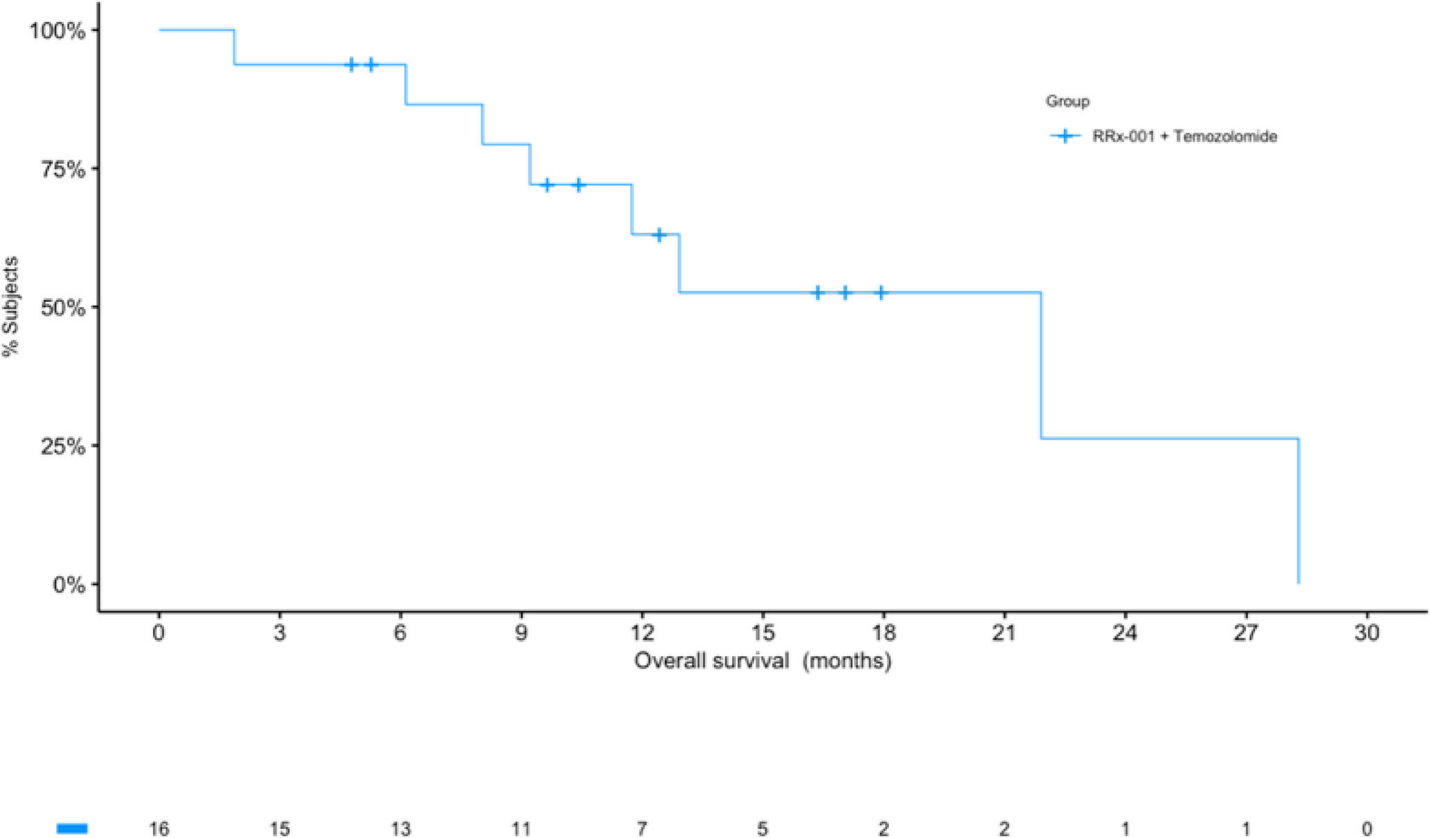
Kaplan-Meier (KM) curves for overall survival (OS). The KM curve for OS shows a median of over 21 months. The number of patients at risk at each timepoint are listed under the x-axis.

From July 2016 through May 2018, 18 patients were enrolled. Demographics and baseline characteristics of these patients are shown below in **Table 1**. 6 patients (33.3%) received at least 6 cycles of treatment (median number, 5 cycles). Tumor progression or death prompted treatment cessation in 2 of 18 patients (12.5%). Kaplan-Meier curves are shown below in **Figure 1**. Toxicity or intercurrent illness did not result in treatment cessation. Two patients withdrew consent.

**Table 1:** Demographics and baseline characteristics.

Treatment: RRx-001 +_ Temozolomide		
Variable	Parameter	Estimate
Age (years)	n	16
	Mean (SD)	59.2 (10.1)
	Median	58.0
	Q1 - Q3	54.8 - 64.0
	Min	37.0
	max	78.0
	IQR	9.2
	Missing	0
Age categories n (%)	(0, 65]	12 (75.0)
	(65, 100]	4 (25.0)
	Total	16 (100.0)
Sex n (%)	Female	11 (68.8)
	Male	5 (31.2)
	Total	16 (100.0)
Race n (%)	American Indian or Alaskan Native	1 (6.2)
	Asian	2 (12.5)
	Black or African American	2 (12.5)
	White	11 (68.8)
	Total	16 (100.0)
Ethnicity n (%)	Hispanic or Latino	1 (6.2)
	Non-Hispanic	15 (93.8)
	Total	16 (100.0)
MGMT Status n (%)	1	9 (56.2)
	2	4 (25.0)
	NA	3 (18.8)
	Total	16 (100.0)
Primary Tumor n (%)	GBM	16 (100.0)
	Total	16 (100.0)
IDHS Status n (%)	1	12 (75.0)
	2	2 (12.5)
	NA	2 (12.5)
	Total	16 (100.0)

NA, Not Available.

## Results

### Primary analysis

**Figure 2:**
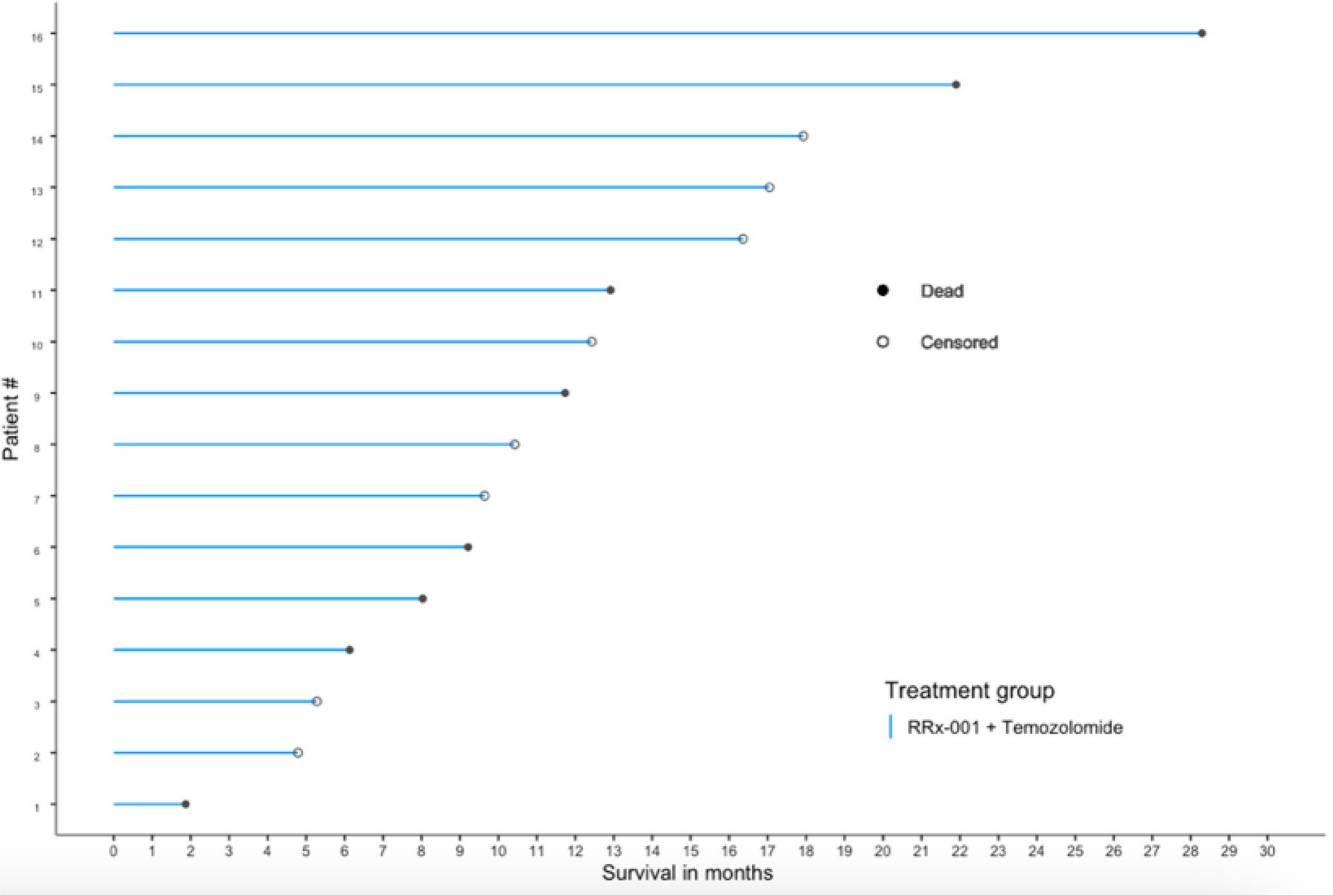
OS swimmer’s plot. This swimmer’s plot shows several patients with extended overall survival.

At the time of analysis (July 23, 2019), 8 of the 16 patients (50.0%) were still alive, with a median follow-up time of 12.5 months (range: 1.9 to 28.3 months (n = 16)). The median overall survival was 21.9 months (95% CI: 11.7 – NA, see **Figure 2**) as compared to 14.6 months reported in Stupp et al. 2017 (see [2]) with radiotherapy plus temozolomide. A swimmer’s plot depicts the length of overall survival segregated by censored/deaths (**Figure 2**). A table of tumor types and overall survival is listed below in **Table 2**.

**Table 2:** List of GBM patients by overall survival.

G-FORCE RRx-001 Overall survival by tumor type			
Subject ID	Primary Tumor	os Months	os Censoring status (1 = Death)
001-001	GBM	28.30	1.00
001-003	GBM	8.03	1.00
001-004	GBM	21.90	1.00
001-005	GBM	12.92	1.00
001-006	GBM	16.36	0.00
001-007	GBM	11.74	1.00
002-002	GBM	17.05	0.00
003-003	GBM	17.93	0.00
003-004	GBM	9.21	1.00
003-005	GBM	12.43	0.00
001-008	GBM	6.13	1.00
001-009	GBM	10.43	0.00
003-006	GBM	9.64	0.00
001-010	GBM	1.87	1.00
003-008	GBM	4.79	0.00
003-009	GBM	5.28	0.00

The overall response rate (ORR) was 18.8% (3/16, 0 CR and 3 PR) and may have been confounded by the development of pseudoprogression, therefore biased. The rate of disease control (stable disease or better) was 68.8% (0 CR, 3 PR, 8 SD)

The median duration of progression-free survival among the 16 GBM patients was 8.0 months (95% CI: 5.0 – NA).

### MGMT status

The MGMT status was prognostic. The median overall survival was not reached for 4 patients with MGMT unmethylated tumors as compared to a 21.9 month median (95% CI: l1.7 - NA) for 9 patients with methylated tumors (3 patients did not have a recorded MGMT status). The median PFS was 6.5 months (95% CI, 4.6 - NA) for MGMT unmethylated tumors and 8 months median (95% CI: 8.0 - NA) for methylated tumors.

The IDH class was also prognostic. The median duration of overall survival was 21.9 months (95% CI, 11.7 - NA) for 12 patients in IDH with Wild type, 12.9 months for 2 patients with IDH Mutant type. The median PFS was 9.0 months (95% CI: 8.0 – NA, n = 12) for patients in IDH with Wild type, 8.0 months (n = 2) for IDH Mutant type. This subgroup analysis is unreliable at best given the severely small subgroups sample size. It is only concluded for completeness, and we draw no conclusion based on the subgroup analysis. Data were unavailable for recursive partitioning analysis (RPA) class.

During the maintenance period there were 6 patients (out of 16 with GMB as primary tumor) who received TMZ + RRx–001 and 10 who received TMZ alone. OS for TMZ alone maintenance was 21.9 months (95% CI: 11.7 – NA) as compared to TMZ + RRx-001 maintenance where the median OS was not reached (95% CI: 6.1 – NA). PFS median for TMZ alone maintenance was 8.5 months (95% CI: 5.0 – NA) as compared to TMZ + RRx-001 maintenance where the median PFS was 8.0 months (95% CI: 4.0 – NA).

### Dose escalation and toxicity

Safety and toxicity data were available as of July 22, 2019, for the 16 (GBM) patients. The most common serious adverse events (SAEs) i.e., those that required hospitalization were hyperglycemia and seizure, occurring in approximately 6.2% (1/16), seizure 31.2% (5/16), headache 6.2% (1/16) and urinary tract infection 6.2% (1/16) of patients. The most common neurological AEs in order of frequency were seizure, headache, and paresthesia (**Table 2**). No SAEs were considered related to RRx-001.

**Figure 3:**
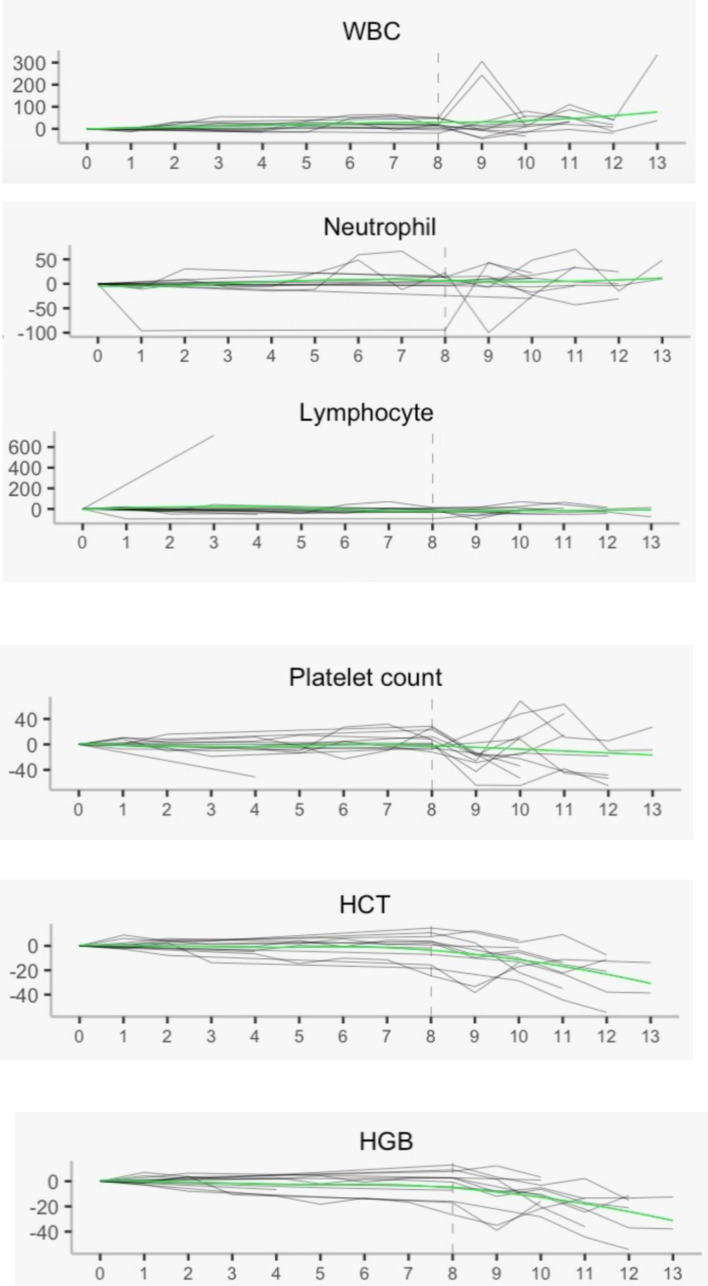
Hematology laboratory measurements with local regression. These measurements show overall stability post treatment with RRx-001, temozolomide, and radiotherapy in all parameters except hemoglobin and hematocrit. WBC, white blood cells; HCT, hematocrit; HGB, hemoglobin.

In addition, RRx-001 appears to attenuate or reduce the decline of white blood cells and platelets but not red cells during treatment with temozolomide and radiation, which possibly indicates partial bone marrow protection as shown in **Figure 3** below. In **Figure 3**, the vertical dotted line represents the delimitation prior to which pre-treatment with RRx-001 was administered. The smooth curve through the scatter plots is obtained with LOESS or locally estimated scatterplot smoother. This LOESS method reveals stable white blood cells (WBCs), neutrophils, lymphocytes, and platelets but not hemoglobin or hematocrit, which dropped well below baseline during treatment with temozolomide after the start of RRx-001.

The rate of severe (grade 3 or more) toxicities were thrombocytopenia 31.2% (5/16) and anemia 6.2% (1/16). The main AEs considered related to RRx-001 were infusion related reaction (25%) and infusion related pain (6.2%).

## Discussion

The standard of care for newly diagnosed glioblastoma with fractionated radiotherapy and temozolomide results in an overall survival of less than 16 months. A single controlled study suggested that TTF (Tumor Treating Fields) can increase survival but the inconvenience of wearing the device, the current lack of confirmatory randomized data, and the skepticism of some practitioners for its overall efficacy has limited the number of patients who use this approach. Clearly, novel agents and treatment strategies to enhance the antitumor activity of radiotherapy and TMZ in the newly diagnosed GBM setting are urgently needed.

RRx-001 is a theoretically attractive agent to add to radiotherapy and cytotoxic chemotherapy based on a preclinical studies demonstrating several potential anti-tumor mechanisms of action including, anti-angiogenesis, nitric oxide donation and vascular normalization, potentiation of radiation-induced reactive oxygen species, NLRP3 inhibition, repolarization of therapy resistance tumor-associated macrophage (TAM). In this G-FORCE-1 trial, RRx-001 plus radiotherapy and TMZ was well tolerated over the entire range of investigated doses with acceptable toxicity. Thus, the primary objective of safety and tolerability was met as this was a Phase 1 trial, which was conducted to evaluate different doses and schedules of RRx-001 in combination with TMZ and radiotherapy.

In conclusion, the addition of RRx-001 to the regimen of radiotherapy and temozolomide was safe and well-tolerated. Also, RRx-001 did not appear to increase hematologic toxicity compared to RT/TMZ alone and may even provide a protective effect. For example, grade 3 or 4 neutropenia was not observed in this small study but had a ≥10% reported incidence in the TEMODAR prescribing information (17). A common ≥ grade 3 reaction of TEMODAR is fatigue (13%), yet in contrast, no grade 3 fatigue was experienced in this study see **Tables 3** and **4**. Protection against neurocognitive decline was not assessed as part of the phase 1 study but will be considered in subsequent clinical trials since RRx-001, which has received FDA orphan designation for the treatment of acute radiation syndrome (ARS), is associated with radio- and chemoprotective properties both preclinically and clinically (18–20). These initial data suggest that RRx-001 may increase RT and TMZ therapeutic tolerability, increasing a subject’s quality of life and decreasing therapeutic toxicity.

**Table 3:** Serious Adverse Events That Led to Hospitalizations.

System Organ Class	Preferred Term	n (%)
Eye disorders	Vision blurred	1 (6.2)
Gastrointestinal disorders	Intestinal perforation	1 (6.2)
	Vomiting	1 (6.2)
Injury, poisoning and procedural complications	Brain oedema	1 (6.2)
	Fall	1 (6.2)
Metabolism and nutrition disorders	Hyperglycaemia	1 (6.2)
Nervous system disorders	Headache	1 (6.2)
	Seizure	5 (31.2)
Psychiatric disorders	Confusional state	1 (6.2)
Renal and urinary disorders	Urinary tract infection	1 (6.2)

**Table 4:** Adverse events of grade 3 or higher correlating with severity.

Adverse Events with Grade ≥ 3		
System Organ Class	Preferred Term	n (%)
Blood and lymphatic system disorders	Anaemia	1 (6.2)
	Leukopenia	1 (6.2)
	Lymphopenia	1 (6.2)
	Thrombocytopenia	5 (31.2)
Ear and labyrinth disorders	Hypoacusis	1 (6.2)
Eye disorders	Vision blurred	1 (6.2)
Gastrointestinal disorders	Intestinal perforation	1 (6.2)
	Vomiting	1 (6.2)
General disorders and administration site conditions	Oedema	1 (6.2)
Injury, poisoning and procedural complications	Brain oedema	1 (6.2)
	Fall	1 (6.2)
Metabolism and nutrition disorders	Hyperglycaemia	4 (25)
	Hypokalaemia	1 (6.2)
Musculoskeletal and connective tissue disorders	Muscular weakness	1 (6.2)
Nervous system disorders	Dizziness	1 (6.2)
	Hemiparesis	1 (6.2)
	Seizure	2 (12.5)
Psychiatric disorders	Confusional state	1 (6.2)
Renal and urinary disorders	Urinary tract infection	1 (6.2)
Vascular disorders	Haematoma	1 (6.2)

Given the promising and novel mechanism of action, further study of RRx-001 in patients with glioblastoma and in a randomized controlled setting (Phase 2) is of interest to inform further clinical development in this indication.

## Data availability statement

The raw data supporting the conclusions of this article will be made available by the authors, without undue reservation.

## Ethics statement

The studies involving human participants were reviewed and approved by Western Institutional Review Board (WIRB) approved this study. The patients/participants provided their written informed consent to participate in this study.

## Author contributions

All authors contributed to the conception and design of the study and participated in the material preparation and manuscript review. NA analyzed the data. BO drafted the manuscript and prepared diagrams. All authors have read and approved the manuscript for publication.
